# Young People’s Experience of a Long-Term Social Media–Based Intervention for First-Episode Psychosis: Qualitative Analysis

**DOI:** 10.2196/17570

**Published:** 2020-06-26

**Authors:** Lee Valentine, Carla McEnery, Shaunagh O’Sullivan, John Gleeson, Sarah Bendall, Mario Alvarez-Jimenez

**Affiliations:** 1 Orygen Parkville Australia; 2 Centre for Youth Mental Health The University of Melbourne Melbourne Australia; 3 Centre for Mental Health Swinburne University of Technology Hawthorn Australia; 4 School of Behavioural and Health Sciences Australian Catholic University Melbourne Australia

**Keywords:** social media, social networking, youth, young adult, psychotic disorders, mHealth, qualitative research

## Abstract

**Background:**

Digital mental health interventions present a unique opportunity to address the lack of social connection and loneliness experienced by young people with first-episode psychosis (FEP). The first generation of digital interventions, however, is associated with high attrition rates. Social media presents an opportunity to target this issue. A new generation of digital intervention has harnessed the popularity of social media to both promote engagement and foster social connectedness in youth mental health interventions. Despite their potential, little is known about how young people engage with, and experience, social media–based interventions as well as the optimal design, implementation, and management needed to ensure young people with psychosis receive benefit.

**Objective:**

This study aimed to explore how young people engage with, and experience, a long-term social media–based mental health intervention designed to address social functioning in individuals with FEP.

**Methods:**

This qualitative study was based on 12 interviews with young people who used Horyzons, a long-term social media–based mental health intervention, as part of a previous randomized controlled trial. A semistructured phenomenological interview guide with open-ended questions was used to explore young people’s subjective experience of the intervention. All interviews were recorded and transcribed verbatim. Data were analyzed using interpretative phenomenological analysis.

**Results:**

A total of 4 superordinate themes emerged during the analysis including (1) shared experience as the catalyst for a cocreated social space, (2) the power of peer support, (3) an upbeat environment, and (4) experiences that interrupt being in Horyzons.

**Conclusions:**

We found that Horyzon’s therapeutic social network fostered a connection and an understanding among young people. It also aided in the creation of an embodied experience that afforded young people with FEP a sense of self-recognition and belonging over the long term. However, although we found that most young people had strong positive experiences of a social connection on Horyzons, we also found that they experienced significant barriers that could substantively interrupt their ability to use the platform. We found that social anxiety, paranoia, internalized stigma, lack of autonomy, and social protocol confusion interfered with young people’s usage of the platform. From a design perspective, digital interventions are flexible and thus equipped to begin addressing these implications by providing customizable and personalized treatment options that account for varying levels of social connection and psychological need that could otherwise interrupt young people’s usage of social media–based interventions.

## Introduction

### Background

The current treatment of psychosis focuses primarily on symptom reduction [1]. Psychological and pharmacological interventions are effective in treating symptoms and preventing relapse to a degree; however, many young people with first-episode psychosis (FEP) experience significant difficulties with regard to social functioning and report high levels of social isolation [2-4]. Despite these findings, very few studies have assessed interventions targeting social functioning as a primary outcome [2,3].

Even in the earliest stages of the disorder, young people with FEP experience reduced social opportunities, have limited social networks, report difficulty developing and maintaining social relationships, and experience high levels of loneliness [1,3,5]. These findings have led to an increased focus on effective interventions that target loneliness and social connection [6]. However, despite the recognized importance of including social connection within a broader recovery framework, there are limited evidence-based solutions available to address this need [3,7,8].

Digital mental health interventions present a unique opportunity to address a lack of social connection and loneliness in FEP [3,8-14]. However, the first generation of digital interventions is associated with high attrition rates [15,16]. Social media presents an opportunity to target this issue. A new generation of digital interventions has harnessed the popularity of social media to both promote engagement and foster social connectedness [8,9,12,13].

### Objective

Despite their potential, little is known about how young people engage with, and experience, social media–based interventions as well as the optimal design, implementation, and management needed to ensure young people with psychosis receive benefit. In addition, little is known about how these interventions are experienced over the long term. This study will address this gap by exploring young people’s experiences of a long-term social media–based intervention to inform the emerging generation of digital mental health interventions. Therefore, this study aimed to explore how young people engage with, and experience, a long-term social media–based intervention designed to address recovery in FEP.

## Methods

### Setting and Design

**Figure 1:**
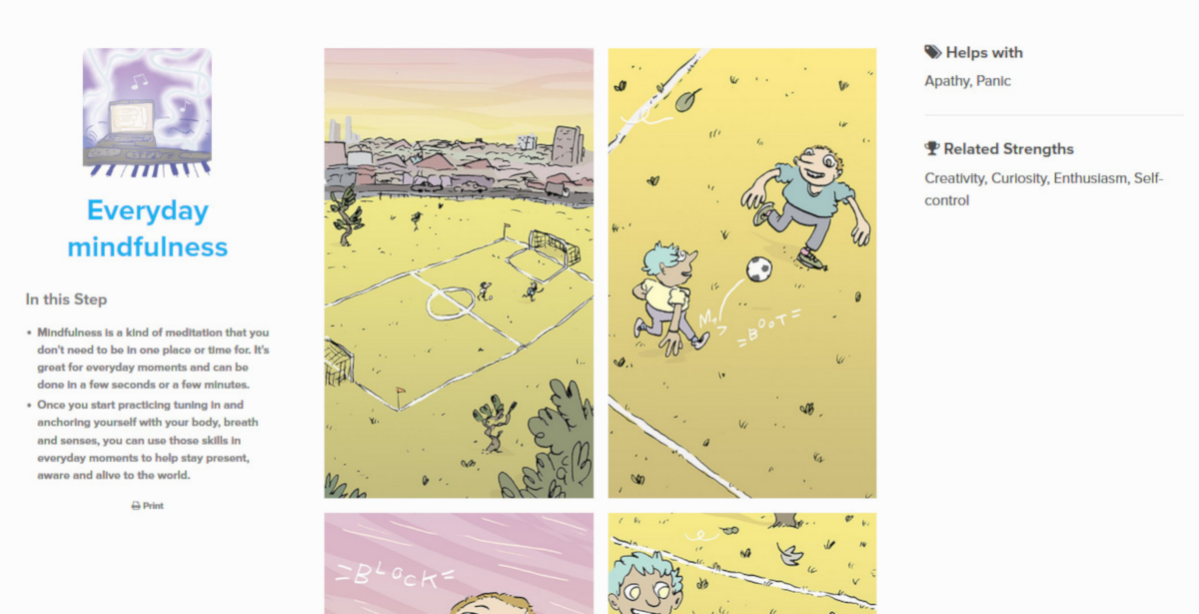
Everyday mindfulness therapy step.

**Figure 2:**
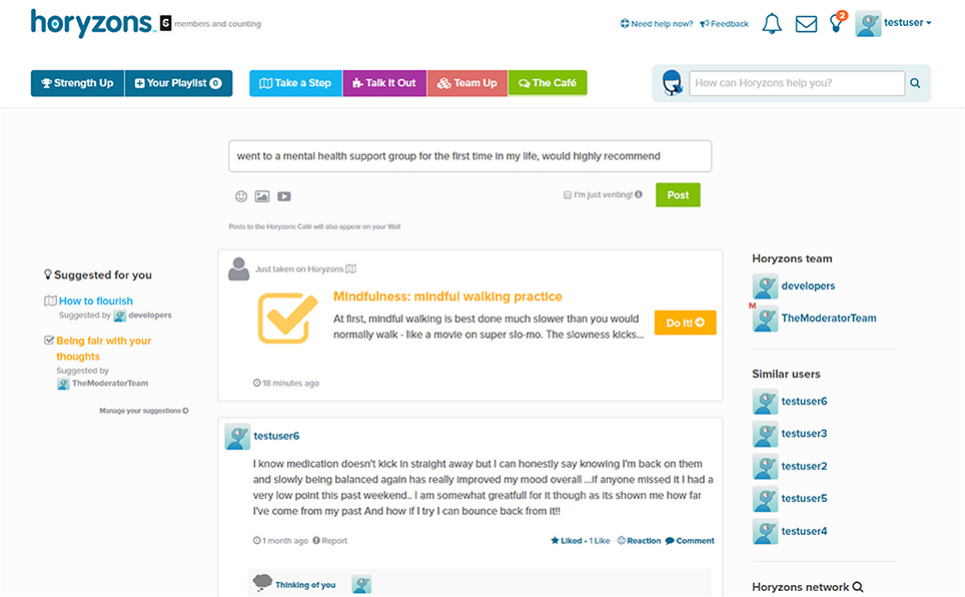
The Horyzons news feed.

This phenomenological qualitative study was based on interviews conducted between October 2018 and March 2019. Young people interviewed in this study had previously participated in the Horyzons randomized controlled trial (RCT; ACTRN12614000009617). The Horyzons RCT evaluated whether a long-term 18-month moderated online social therapy (MOST) platform, known as Horyzons, was superior to 18 months of regular care, following discharge from 2 years of treatment at an FEP early intervention service [8]. The MOST platform was co-designed and developed by a multidisciplinary team of peer workers, clinical psychologists, computing and information systems researchers, software developers, illustrators, and writers [17]. Horyzons was underpinned by a strengths-based and positive psychology framework designed to generate long-term functional recovery and increase well-being in FEP. It was a closed digital platform with options to engage in web-based therapy pathways (Figure 1), speak with peer and clinical moderators (both privately via a web-based chat and publicly on the social network), and connect with other young people via the therapeutic social network, which was referred to as the *newsfeed* (Figure 2) [8]. The digital functions of the platform, such as the web-based therapy pathways, were reinforced by human support provided by the clinical moderators through a supportive accountability model [18]. This contact was primarily provided through web-based chats and phone calls. A key clinical moderator was also allocated to each participant and was responsible for overseeing their engagement and progress over the span of the intervention [18]. Young people could connect on the social network by posting and commenting, sharing experiences, and giving and receiving support. Conversation could occur between participants in a freeform way on the newsfeed and they could also take part in focused group discussions through a feature referred to as *Talk It Outs* [8]. These discussions were overseen by peer moderators. As described in the Horyzons protocol, the functions of the platform were “designed to reinforce each other, creating a flow for the young person between the social and therapy elements” [8]. For the Horyzons RCT, a total of 170 young people approaching or recently discharged from the Early Psychosis Prevention and Intervention Centre in Melbourne, Australia, were recruited. Of the 170 participants, 85 were randomly allocated to the specialist treatment group. This gave them access to the Horyzons platform for an intervention period of 18 months. The remaining 85 participants continued with treatment as usual.

### Participants

The study inclusion criteria were as follows: (1) participation in the Horyzons RCT, (2) randomization to the treatment group of the Horyzons RCT, and (3) completing the Horyzons RCT. With the aim of sampling a range of young people with different levels of platform usage [19], a quantitative categorization of Horyzons participants was conducted. This categorization was based on each participant’s overall number of log-ins to the Horyzons platform. Thus, all 85 participants randomized to the treatment group of the Horyzons RCT were divided into 4 quartiles based on the number of overall log-ins to the platform over the 18-month intervention period. Overall, log-ins ranged from 1 to 1568. The number of log-ins associated with each usage group is detailed in Table 1. The quartiles represented 4 usage groups: very low, low, moderate, and high usage. Young people from each usage group were then randomly contacted via phone call or text and invited to participate in a qualitative interview.

**Table 1:** Log-ins, mean, and standard deviation associated with each usage group.

Usage group	Total (N=84), n (%)	Log-ins	Mean (SD)
Very low	21 (25)	1-8	5 (2.2)
Low	21 (25)	9-23	15 (4.9)
Moderate	22 (26)	24-80	31 (19.3)
High	20 (24)	88-1569	366 (405.7)

### Data Collection

All interviews were conducted using a phenomenological approach [20]. This interview method is concerned with the lived experience of a phenomenon, as experienced subjectively by the individual [21]. The interview schedule comprised 2 extremely broad, open-ended questions: (1) What was Horyzons? and (2) What was Horyzons like? These questions were designed to elicit young people’s experiences of the Horyzons platform without leading them in a particular direction. Subsequent prompts were used if necessary; however, the method calls for the interviewer to follow the leads presented by the participant. Participants were given the option of completing the interview at the Orygen clinic, home, or in a public place (eg, library or café). Subsequently, interviews were conducted in a range of locations. Before beginning each interview, participants were required to complete a plain language consent form and given the opportunity to ask questions. Author LV conducted all interviews and had established relationships with all participants due to a previous position as a research assistant on the Horyzons RCT. LV is an early career researcher with extensive quantitative interviewing experience and is currently undertaking a research project using phenomenological methodological interviewing. Field notes were taken during the interviews, and a reflective log was maintained over the interview period. In accordance with the interpretative phenomenological analysis (IPA) approach, data collection, transcription, and preliminary analysis were conducted in parallel [22]. All interviews were audio-recorded and transcribed verbatim. All participants were reimbursed Aus $20 (US $12.63) for their participation in the study. All young people approached to participate in the study were interviewed with the exception of one young person who became distressed at the beginning of the interview due to acute psychotic symptoms, and the interview was quickly terminated. The recruitment of participants continued until a saturation of themes was reached at 11 participants [20]. The twelfth participant was then sampled to ensure that no additional themes emerged [20].

### Data Analysis

Interviews were analyzed using IPA. In accordance with IPA, analysis for the study commenced once the first qualitative interview had been completed and transcribed [21]. Interviews were analyzed individually and in relation to all other interviews completed over the course of the study in a multilayered and overlapping IPA framework. Themes were derived directly from the data collected. Given that interpretation can be based on the context in which the phenomenon is viewed, member checking was not offered to participants because of the interpretative nature of the analysis used [22]. To begin, multiple close readings of each transcription were conducted. Initial responses to the data were noted and recorded. Codes or brief summaries describing the data were recorded. These codes formed the bases of emerging themes and patterns across the overall data set. The identification of reoccurring themes within and across interviews emerged and was noted. Some themes were distinct from one another, whereas others merged as superordinate themes. Strategies to maintain rigor, reliability, and validity throughout the analysis process included prolonged engagement with the data and thick and rich descriptions of results [23]. Author LV conducted the analysis and engaged in regular reflexive discussion regarding the codes and subsequent themes with the senior author (SB) [23]. Microsoft Excel was used in the data analysis process.

## Results

A total of 12 participants aged 19 to 28 years (mean 23 years) were included in the study; 7 of the participants were female and 5 were male. All of the female participants were cisgender. Of the male participants, 4 were cisgender and 1 was transgender. Multiple participants from each of the 4 usage groups were interviewed. Interview times ranged from 30 to 86 min. The time period in which participants were interviewed for this study following the last date they were able to access the Horyzons platform ranged from 1 to 5 months. The mean follow-up time was 3 months. All participant names were replaced with an alias to ensure privacy. Table 2 gives a description of participant characteristics.

In total, 4 superordinate themes emerged during the analysis, with each theme divided into subthemes to comprehensively capture the phenomenon explored. The 4 superordinate themes were (1) shared experience as the catalyst for a cocreated social space, (2) the power of peer support, (3) an *upbeat* environment, and (4) interruptions to *being in* the Horyzons space.

**Table 2:** Descriptive summary of participant usage groups, alias, gender, age, follow-up time, length of interview, and overall log-ins to the Horyzons platform.

Usage group and alias		Gender	Age (years)	Time since last possible log-in (months)	Interview length (mins)	Overall log-ins, n
**High**						
	Sam	M^a^	28	2	49	1568
	Babak	M	23	3	25	109
	Audrey	F^b^	24	5	20	1230
**Moderate**						
	Emily	F	26	4	49	66
	Jacob	M	21	5	50	65
**Low**						
	Tom	M	22	1	86	18
	Celeste	F	23	3	58	12
	Olivia	F	22	4	79	13
	Abigail	F	19	5	32	23
**Very low**						
	Linh	F	25	2	56	6
	Eugene	M	25	2	42	8
	Ruby	F	20	3	45	7

^a^M: male

^b^F: female.

### Shared Experiences as Catalysts for a Cocreated Social Space

#### Horyzons Was a Place and “I Felt at Home”

Young people used language commonly associated with the description of physical places when describing experiences of the Horyzons platform. That is to say that they spoke of Horyzons as a tangible or an embodied space that they could *arrive at* and *be in*. For instance, Audrey’s description of sharing accomplishments with her peers on the therapeutic social network evokes imagery of *traveling to* and *being at* a physical destination:

So, I went there, and I would talk, talk about my achievements. I felt proud of myself for doing something. People would encourage me or commend me, yeah. So, it was a nice place to be.

Audrey

Audrey uses the spatial and social descriptors “went there*,”* *“*talk about*,”* *“*people would encourage me*,”* and “a nice place to be*”* to describe moments that are technically confined to a computer, tablet, or phone. However, her phrasing of these experiences points toward a perception of Horyzons as a physical experience. Instead of detailing how she *uses* the platform, Audrey describes an experience of actually *going there* and *being in* the Horyzons social space.

#### “Don’t Necessarily Know Each Other Personally” But a Connection Through Shared Experience

A number of participants described an experience of *knowing* other members of the platform. Interestingly, however, the *knowing* in most cases did not equate to a direct social relationship or connection, either physically or digitally, but was instead based on the participants’ *common* history of mental health experiences:

It's like… war veterans… where you have all the gatherings of people who have been through wars and they don’t necessarily know each other personally, but they’ve all got that common war experience. And they can relate to each other and they know what they’ve been through.

Sam

Sam’s description builds upon commonplace social definitions of *knowing* and *connection.* It offers an alternative construct of what it might be to *know* other members in the particular place that is the *Horyzons space*. In this space, *knowing* each other may not be contingent on direct social contact but instead on the concept of *understanding* and *relating* to the *common* experience of being a young person with lived mental health experience.

#### “I Just Felt Like Relieved That I Wasn't The Only One”

Young people detailed a range of positive emotional responses at not being *the only one* with an experience of psychosis and other mental health experiences on the Horyzons platform.

*Being in* this space with others who shared this *common* history had a strong impact on participants. Many expressed that they could *relate* to others and both *understand* and feel *understood* in this environment. The sharing of experiences on the Horyzons platform by group members had a normalizing impact for some participants. For instance, Sam expressed:

It helped sort of reinforce that some of my experiences and feelings are normal… I’m not a weirdo.

Sam

Some members found it easier to share their own experiences with the group given they were in a shared social space with others who also had a lived experience of mental ill health:

My story is not as twisted as it is common, shit that happens. I could feel more comfortable sharing knowing that it’s common...

Jacob

For some, sharing their personal stories with the group was a therapeutic activity in itself. The act of sharing supported the young person to unpack and reflect on their mental health experiences:

I wanted to support them, so I think I reflected on what I have been through…Actually gave them my experience, how I fought through it.

Emily

For Olivia, the shared experience of mental ill health sparked a sense of pride, pride that the group existed despite the adversity that its members face:

For me, it was just really important to see that there were other people that were kind of just saying like,’ yeah, I’m proud.’… I think it’s really important to be proud even though you’ve got fucking mental problems.

Olivia

Some participants identified the benefit of learning about other people’s experiences, even if their stories were not directly relevant to the young person’s own life. This information was a way to feel connected to others, receive encouragement, and access a diverse range of coping methods:

We didn't always have the same illness or anything. It was nice to hear people, people's experiences. It was encouraging to hear, yeah...Um, just how they dealt with the problem...And everyone approached them differently.

Abigail

One particular participant identified other members on the Horyzons platform that he knew from offline spaces. For him, this social overlap helped to reinforce that “mental health is a common thing*.*”

For some participants, the opportunity to share a space with peers that they were able to relate to, and understand their experiences, reminded them of painful memories when their mental health struggles had been minimized, misunderstood, or mocked:

When I was with Orygen I told them the first time what had happened [family], and they're like “nah it's not true, it's just all in your head, it's all in your mind”, and they didn't believe me, and it kinda just felt like I couldn't trust them.

Emily

#### “A Barrier That’s Automatically Broken Down”

As described in the themes above, the cocreation of space and the concept of a *common* mental health history came together to create a social environment on Horyzons that participants described as *more intimate* than other spaces*.* Many participants described a greater sense of ease on the platform between peers than in other social spaces, both on the web and offline. This ease between young people acted to break down conventional social barriers and accelerated bonding:

Whereas I guess with traditional social media you don’t just go up to random people and say “hey I’ve got this thing, do you want to talk about it?” whereas on Horyzons it was sort of specially crafted for that sort of thing, so it was a lot easier to approach people and sort of form bonds and stuff.

Sam

For participants, the awareness that they were among others with a shared experience meant they felt less judged and as a consequence of this, more relaxed to share and connect:

I connected to them a lot more easier than I would with others… I felt like I was in a more calm relaxed space connecting with them than other people that I meet at Uni… Like they were more open, non-judgemental... I felt relaxed…Yeah I felt really relaxed.

Emily

This dynamic appears to be somewhat cyclical in nature for participants. The recognition that their peers have a shared experience and a *more open* and *nonjudgmental* approach results in a more *relaxed* environment conducive to sharing. Once this process has occurred, several young people described an experience whereby they saw others sharing and this created a sense of positive responsibility to their peers in which they described wanting to “own up a bit more” to the group:

They were pretty much open with what they were going through…and I thought to myself ‘I should open up at least and see where I would go’…Just like take a shot at it.

Emily

### The Power of Peer Support

#### “Don‘t Really Know Unless You’ve Experienced It”

In the Horyzons context, the function of offering and receiving support was generally operationalized through public posts and replies on the social network. Participants expressed that offering and receiving advice and support from others with lived mental health experience, otherwise known as peer support, was a valuable practice that is unique to the peer-to-peer relationship. This dynamic is situated within the context of shared mental health history, a history that a clinician may not always have direct experience with. In the words of Abigail:

They don't really know unless [they’ve] experienced it.

Abigail

Like peer support really is helpful for me in particular, just cos like, I kinda find it more difficult to open up to someone that doesn’t really fully get the picture, even if they’re like a completely trained professional.

Ruby

Participants communicated that their life experiences should position them at the center of the Horyzons platform. They identified that their mental health journey can help to guide current day problem solving for themselves and others:

Apart from starting it and summarizing it at the end [group discussions], I feel like it was mostly handed over to the users, which is probably a good thing because it allows the people with the experiences to sort of come up with their own ideas and thoughts.

Sam

For some young people, the advice of a peer is elevated above that of a “completely trained professional*”* or *“*someone giving a TED Talk*.”* Some young people expressed that support from a person with lived experience would be easier to implement in their own life because that individual would “fully get the picture” and be more likely to offer *“*practical” and *“*realistic” advice:

I want to know what real everyday people do. Not a social media presence person, just the person on the other side of the road. What do they do? ...It might not work for me…but it's probably most interesting because it's probably most um, uh, doable?

Celeste

#### “I Love Being a Massive Resource for That Sort of Thing”

Many participants described offering support and advice to others based on knowledge gained via their own mental health experiences as an empowering, validating, and, at times, joyful function of the Horyzons platform. Emily expressed, “I felt really calm, relaxed and over the moon because I was able to help someone.”

#### “Responsibility to… Basically Just be Their Secondary Psychologist”

In total, 2 participants expressed strong reservations regarding what they experienced as an unwanted obligation to provide peer support to other members on the platform. Ruby stated, “just that fear if someone messages me, I have a responsibility to talk to them, to support them, to basically just be their secondary psychologist.”

Ruby describes experiencing a lack of control over the peer support function of the platform, “I don't always like it when people open up to me when I don't ask for it.” As well as anticipatory fear that denying a request for peer support from another member would result in a negative interaction, “I was scared that they’d like start cyber-stalking me or something.”

### An “Upbeat” Environment

As mentioned earlier, a positive psychology framework underpinned the Horyzons RCT. This framework informed how clinicians moderated the platform. For example, using a strengths-based reframing approach to young people’s posts. Participants reported a spectrum of emotional responses to the environment that this clinical approach created on the Horyzons social network.

On one end of the spectrum, Celeste described feeling frustrated and patronized by this approach and described the social network as *“*mind-numbingly positive.” She argued that this type of environment impedes the process of sharing the *“*bad stuff*”* that young people living with mental health problems can experience. To discuss difficult topics, which Celeste identified as an important process of mental health recovery, members would need to go against the grain of the established *“*upbeat” environment.

Celeste described feeling alienated by this approach and went so far as to describe a phenomenon akin to an uncanny valley experience. That is to say, the platform looked similar to other social networks, but that the environment did not feel like any other social spaces she was accustomed to. This irregularity led to a feeling of unease:

It was just odd, and it didn‘t feel like it could be genuine because it's not my experience of how people interact with each other in the everyday world, you know? [Celeste] 

In contrast to Celeste’s experience, Audrey identified the *“*positive” environment on Horyzons as an important element of her high usage level. She did not want to spend time at a place where “the people are all negative*.”*

Olivia’s experience was similar to Celeste’s in that she experienced the *“*upbeat” nature of the platform as different from that of commercial social network sites (SNS). However, she also identified that although it was positive for people with mental health difficulties to connect with others in certain mental health pockets of social media, such as “Instagram,” certain relationships and dynamics that are borne from these unregulated digital spaces can have negative impacts on the young people who frequent them. For example:

You know, go on Instagram-... type in eating disorder, you got a whole world there. You know? You can type in depression; you've got a whole world of people struggling. And it's a good and a bad thing. So, it's good as in they can connect with other people. But, it's bad as in you get triggered constantly. Rather than that, Horyzons was, “Yeah, we're all struggling... but this is a place for improvement.”

Olivia

For Eugene, the experience of being in an environment with a psychological lens tarnished his experience of the social network. He said:

…that’s what my brain tells me, it’s just like, super wanky. Like, I think psychology and… that sort of stuff is really wanky.

Eugene

He did, however, describe experiencing something of a psychological shift when the platform was able to support him during a time when he was experiencing *“*major issues*.”* As a result, he identified that Horyzons *“*may have a point” and may not be *“*so wanky*.”* A shift, such as a change in mind or need, was a phenomenon reported by other participants in reference to a range of issues on the platform.

### Experiences That Interrupt Being in the Horyzons Space

Participants identified a lack of motivation to participate in the social network as well as a range of experiences that interrupted their ability to *be in* the Horyzons social space in an immersed or *relaxed* way. These interruptions gave way to a self-conscious or difficult moment, or a series of moments, which created tension between the young person and their ability to use the platform. Participants identified that these incidents could occur before, during, or after single sessions and throughout the overall intervention experience itself. Some participants identified multiple interruptions, or ruptures, throughout their Horyzons experience.

#### Absence of Motivation

A number of participants described an absence of motivation to participate in the social network, either before any initial engagement with the platform or at some point during the intervention period. This was due to the personal assessment that the social network was not relevant to them and their needs.

For instance, Tom identified that from the beginning of his intervention period, he intended to engage solely with the psychosocial content of the platform and expressed an active disinterest in the social network. He described experiencing ample levels of social media in his daily life and was not interested in getting *“*bogged down” in that aspect of the platform.

Jacob identified that although Horyzons was valuable during the first half of his intervention period, he went on to experience a *“*tipping point” in which he felt he had moved beyond what the platform was able to offer him. He explained, “that’s not the stage of life that I'm at anymore*.*”

#### Experiences That Interrupted Being in the Horyzons Space

A number of young people identified paranoia, social anxiety, and internalized stigma as experiences that interrupted them from *being in* the Horyzons space. These types of interruptions generally occurred *during* the young person’s engagement with the platform. However, while they were attempting to engage, an emotional or psychological barrier arose that impeded that engagement. This ultimately took them out of the immersed or *relaxed* experience of *being in* Horyzons.

Paranoia in relation to Horyzons itself and paranoia in relation to social media use more broadly was a phenomenon described by 2 participants. Emily described experiencing paranoia in relation to social media use more generally, whereas Olivia expressed experiencing disruptive paranoia in regard to the Horyzons platform directly. For instance, she said, “I just get really paranoid about like who's behind this and stuff like that…like what are the motives behind this, and stuff like that*.*” This experience of paranoia interrupted Olivia’s capacity to share candid information and engage directly with other members on the platform. The act of even logging in to the site was difficult, as she explained, “They’re watching me too closely. They know too much.”

Participants also described experiencing social anxiety related to the complex psychological process of posting in the social network. This anxiety is generally related to how others might perceive them and prevented many young people from posting on the platform. For example, Ruby expressed fear of overwhelming others with the content of her posts and wrote and deleted *“*5 to 10*”* messages that she ultimately never shared with the group. Babak felt that his thoughts may not be important enough to share. A number of participants expressed uncertainty about Horyzon’s general social protocol and speculated that they were concerned about breaking *“*weird, unwritten rule*s*” that they were not aware of.

Multiple participants described feeling nervous that if they were to post, then other members may not respond, or may not respond in a desired way. For instance:

It would just sit there, and no one would respond to it...and then I’d be like “ah ok” I’m just kind of in an echo chamber [laughs]...so embarrassing … [interviewer: how come?] cos like no one would really care. It would just kind of sit there and look weird. It’s like having a spotlight on yourself and everyone just staring at you and it’s completely silent.

Ruby

This idea of *sharing anxiety*—that is, the phenomenon of experiencing anxiety at the prospect of posting a message to the social network—interrupted young people’s ability to act spontaneously in the social space. They reported feeling frozen in moments of rumination about possible negative consequences of posting that could go on for a short time, such as minutes, or could impede them from posting on the platform throughout their whole Horyzons experience, despite identifying that there was content they would like to share with other members. Interestingly, however, all but one participant who reported experiencing sharing anxiety about posting but went on to post did not go on to report any negative consequences of posting. On the contrary, Audrey reported that although replies to her posts in the Horyzons space were slower than she was accustom to on other social networks, she described feeling *“*patient” while waiting a *“*day or two” for a reply and felt *“*excited” when she received a response. Furthermore, she felt *“*happy” to receive fewer comments on Horyzons than compared with other social media because she considered the replies received on Horyzons to be genuine and *“*not just for attention.”

However, one particular participant expressed that he continued to think about a post he shared on the network many months after he originally posted it. After initially sharing the post, Jacob became concerned that a moderator had potentially “hidden” it from the public feed because of the possibility that it was inappropriate:

I don't know if this is true or not, but I don't know if a moderator actually hid the post for a bit… I'm not sure… I didn't write again, because maybe then I started second thinking the post, maybe it wasn't appropriate.

Jacob

This confusion transformed into anxious rumination that interrupted Jacob’s ability to post again during his intervention period. This may also be related to his decision that he had reached a *“*tipping point” and no longer needed the site.

Eugene experienced engaging with the Horyzons platform as incongruous with his sense of self. He considered himself older, more independent, and more advanced in his career than the other members of the platform and as a result, he felt an internalized pressure that he *“*shouldn’t need this*.”* Eugene’s social struggle to engage with the platform was also closely tied to the judgments he was concerned his colleagues would make of him. He was concerned that they would not “trust” him professionally if they were aware of his mental health history. Eugene appeared to internalize this stigma and his ability to access support, even when identified as needed, was interrupted.

## Discussion

### Principal Findings

To the best of our knowledge, this is the first study to qualitatively explore young people’s experiences of using a long-term social media–based intervention in FEP recovery. In relation to social connection, we found that experiences could be grouped into 4 superordinate themes, as detailed above. These themes have the potential to inform the development and implementation of social media–based interventions. We found that (1) peer support on a therapeutic social network could be beneficial and engaging for some, and burdensome for others; (2) the number of log-ins did not neatly reflect a young person’s experience of the intervention, (3) unclear social protocol created an uncomfortable social environment in the digital space, and (4) social anxiety, paranoia, internalized stigma, and the perception of limited autonomy could interrupt a young person’s ability to engage with the platform.

In line with previous findings, results from this study indicated that many participants lacked a confidant in their everyday life, a friend with whom they could confide in regarding their mental health experiences [1]. We found that the therapeutic social network offered participants a much-needed opportunity to feel heard, understood, and supported by their peers. For some, this was the first time they had felt connected to others in this way. These findings support previous research that has identified the value of peer support practice in mental health [24,25]. The supportive dynamic between peers on Horyzons aided in the creation of an embodied space in which young people experienced an ongoing sense of belonging. This study found that young people place great value on the advice and support offered by their peers, at times above that of a trained mental health professional. Some advised that they were more likely to activate behaviors suggested by their peers than those of a clinician. This example emphasizes the powerful role that peer support can, and does, play in shaping young people’s mental health recovery. It also highlights the instrumental way peer support can create a digital environment that is (1) an engaging pathway to mental health treatment and to the web-based intervention itself and (2) a potentially therapeutic dynamic in itself.

In addition, this research also found that it was possible for young people to experience anxiety, to experience burden, and to perform avoidant behaviors at the perceived expectation to provide peer support to others on the web. This example illustrates the varied needs among young people (ie, what some find helpful, others may find burdensome), as well as the need to consider the autonomy of the user when designing and implementing interventions. This finding highlights the significance of recognizing young people as individuals with individual needs. Fortunately, the mode of digital intervention is well equipped to address this implication by providing customizable treatment options that are applicable to very large groups of diverse individuals.

We found that young people have varying levels of interest with regard to social connection on the web. Therefore, designing customizable interventions that allow young people to identify the importance of this function in regard to their own mental health treatment journey is recommended. For example, personalized onboarding options empower young people to determine the relevance of digital intervention functions, such as the level of peer support they wish to engage with. This places more power in the hands of the user and, in accordance with self-determination theory [26], has the potential to strengthen their engagement with the intervention overall. Furthermore, personalization should be temporally flexible, as this study also found that young people’s mental health needs were dynamic in nature and could change over the course of a long-term intervention.

The knowledge that young people had internal experiences that interrupted their ability to be *in* the Horyzons space was a novel finding that has not been previously explored. By asking young people about their experiences of using the platform, we were able to elicit internal barriers to use. Social anxiety, level of paranoia, internalized stigma, and lack of perceived autonomy in providing peer support actively interrupted young people’s ability to remain in the Horyzons social space. In line with previous findings that social anxiety is a significant health issue for young people experiencing FEP, this study found that many participants described experiencing social anxiety when using the Horyzons platform [11]. We suggest that anticipating and accounting for these experiences in the design and implementation phase of a digital platform could potentially enhance a young person’s ability to use social media–based interventions. This study found that experiences of social anxiety in the digital therapeutic space may differ from that of offline social anxiety, or even anxiety in commercial social media spaces, such as Instagram and Twitter. For example, fear of overwhelming peers with the content of a mental health post may be unique to the therapeutic social network space. As such, young people may experience a new phenomenon of *therapeutic social media anxiety*. As such, anticipating and attempting to circumvent these experiences may increase young people’s level of engagement and/or benefit from social media–based interventions. On the basis of these findings, we suggest 3 tangible approaches to addressing digital social anxiety in the therapeutic social network space: (1) As discussed above regarding the personalization of web-based peer support, offer young people the power to customize their level of engagement with others. (2) Pre-empt and creatively account for prevalent digital social anxieties in the design phase of an intervention. For instance, work to normalize the experience of posting anxiety by signposting this as a common fear through comics and quotes from previous users, this may work to normalize and reduce hesitations experienced by the user. (3) Future research should focus on the design of a tool that measures these types of engagement interruptions.

In line with previous findings [27], the results from this study indicate that the level of usage, in this case, the number of log-ins, does not neatly align with a young person’s level of expressed connection with the platform. For instance, young people categorized as low or very low users still contributed to themes that characterized Horyzons as a supportive, connected, and embodied experience. The more limited use of the platform appeared to be driven less by indifference or dislike for the intervention itself and more by young people’s complex and dynamic social and psychological experiences that interrupted their use of the platform. For example, social anxiety, internalized stigma, level of paranoia, and previous experiences providing peer support were some of the overarching themes that interrupted usage and were related to lower log-ins on the platform. Most young people had strong positive experiences of social connection on Horyzons. Importantly, however, the majority also experienced barriers to engaging with the platform. That is to say, the helpfulness of Horyzons was considered high, but so were the barriers.

This study found that designing a therapeutic social media–based intervention, such as Horyzons, in a similar way to existing commercial SNS archetypes, such as Facebook or Instagram, could create a sense of unease or discomfort for participants due to conflicting social protocols. For example, in many ways, Horyzons felt similar to commercial social media, but there were also distinct differences felt by participants with regard to the different social norms of the platform. For instance, Horyzons encouraged supportive engagement between members. By comparison, a similar act of responding to a personal post on a stranger’s page on Facebook could be considered a social transgression. This highlights a nuanced misalignment between therapeutic and commercial social media protocols. In other words, Horyzons looked and functioned similarly to Facebook, which for participants evoked a Facebook-like behavioral framework. However, the social protocol encouraged by Horyzons did not exactly reflect Facebook’s social protocol. As a result, young people reported feeling concerned or anxious about performing suggested tasks on Horyzons for fear of behaving inappropriately socially. This finding is in line with previously identified concerns [28] that digital mental health services are at risk of engaging in *psychological skeuomorphs* or the danger of designing derivative models of care and in doing so miss the opportunities that come with reimagining virtual models of care. In some ways, that Horyzons felt similar to commercial SNS may have led to higher user engagement because it is popular and compelling to young people. However, it may have also led to young people experiencing social anxiety or feeling socially exposed due to the nature of the content (ie, sharing mental health experiences in an arena that is not typically experienced in this way). Reimagining how social media–based interventions could still be compelling for young people due to their popularity but feel safer or more socially comfortable to participate in these spaces could be a possible way to reduce internal barriers to engagement. Thus, further research on both features and measures that can enhance engagement or reduce interruptions to a young person’s experience of social media–based interventions over the long term would be of interest.

### Strengths and Limitations

Recruiting a large sample for the original Horyzons RCT contributed to wide variation in platform usage. Thus, for this study, we were able to purposefully sample a diverse representation of young people from a variety of usage groups with the aim of exploring different user experiences that may have been associated with the level of usage. Although we found that low usage did not result from a blanket disinterest in the platform, this variety in sampling allowed for a better insight into the internal experiences that both drove and hindered usage on the platform from a variety of users. Additionally, this is the first study to explore an 18-month intervention period and as such afforded us the opportunity to explore adherence, experiences, and internal barriers to use over time, which had not yet been previously explored for this length of duration. With regard to limitations, recall bias could have impacted the participant’s ability to discuss their experience with the intervention. It could be beneficial to interview participants closer to the completion of their intervention time or in accordance with user design theory, which may suggest interviewing in parallel with the intervention.

### Conclusions

We found that Horyzon’s therapeutic social network fostered connection and understanding between young people. It also aided in the creation of an embodied experience that afforded young people with FEP a sense of self-recognition and belonging over the longer-term. However, although we found that most young people had strong positive experiences of social connection on Horyzons, we also found that they experienced significant barriers that could substantively interrupt their ability to use the platform. We found that social anxiety, paranoia, internalized stigma, lack of autonomy, and social protocol confusion interfered with young people’s usage of the platform. From a design perspective, digital interventions are flexible and thus equipped to begin addressing these implications by providing customizable and personalized treatment options that account for varying levels of social connection and psychological need that could otherwise interrupt young people’s usage of social media–based interventions.

## Abbreviations

FEP: first-episode psychosis
IPA: interpretative phenomenological analysis
MOST: moderated online social therapy
NHMRC: National Health and Medical Research Council
RCT: randomized controlled trial
SNS: social network site

## References

[1] da Rocha BM, Rhodes S, Vasilopoulou E, Hutton P (2018). Loneliness in psychosis: a meta-analytical review. Schizophr Bull.

[2] Santesteban-Echarri O, Paino M, Rice S, González-Blanch C, McGorry P, Gleeson J, Alvarez-Jimenez M (2017). Predictors of functional recovery in first-episode psychosis: a systematic review and meta-analysis of longitudinal studies. Clin Psychol Rev.

[3] Lim MH, Gleeson JF, Alvarez-Jimenez M, Penn DL (2018). Loneliness in psychosis: a systematic review. Soc Psychiatry Psychiatr Epidemiol.

[4] Cotton SM, Lambert M, Schimmelmann BG, Filia K, Rayner V, Hides L, Foley DL, Ratheesh A, Watson A, Rodger P, McGorry PD, Conus P (2017). Predictors of functional status at service entry and discharge among young people with first episode psychosis. Soc Psychiatry Psychiatr Epidemiol.

[5] Sündermann O, Onwumere J, Kane F, Morgan C, Kuipers E (2014). Social networks and support in first-episode psychosis: exploring the role of loneliness and anxiety. Soc Psychiatry Psychiatr Epidemiol.

[6] Lim MH, Gleeson JF, Rodebaugh TL, Eres R, Long KM, Casey K, Abbott JM, Thomas N, Penn DL (2019). A pilot digital intervention targeting loneliness in young people with psychosis. Soc Psychiatry Psychiatr Epidemiol.

[7] Alvarez-Jimenez M, Gleeson J, Bendall S, Penn D, Yung A, Ryan R, Eleftheriadis D, D'Alfonso S, Rice S, Miles C, Russon P, Lederman R, Chambers R, Gonzalez-Blanch C, Lim M, Killackey E, McGorry P, Nelson B (2018). Enhancing social functioning in young people at ultra high risk (UHR) for psychosis: a pilot study of a novel strengths and mindfulness-based online social therapy. Schizophr Res.

[8] Alvarez-Jimenez M, Bendall S, Koval P, Rice S, Cagliarini D, Valentine L, D'Alfonso S, Miles C, Russon P, Penn DL, Phillips J, Lederman R, Wadley G, Killackey E, Santesteban-Echarri O, Mihalopoulos C, Herrman H, Gonzalez-Blanch C, Gilbertson T, Lal S, Chambers R, Daglas-Georgiou R, Latorre C, Cotton SM, McGorry PD, Gleeson JF (2019). HORYZONS trial: protocol for a randomised controlled trial of a moderated online social therapy to maintain treatment effects from first-episode psychosis services. BMJ Open.

[9] Alvarez-Jimenez M, Alcazar-Corcoles M, González-Blanch C, Bendall S, McGorry P, Gleeson J (2014). Online, social media and mobile technologies for psychosis treatment: a systematic review on novel user-led interventions. Schizophr Res.

[10] Lal S, Nguyen V, Theriault J (2018). Seeking mental health information and support online: experiences and perspectives of young people receiving treatment for first-episode psychosis. Early Interv Psychiatry.

[11] McEnery C, Lim MH, Knowles A, Rice S, Gleeson J, Howell S, Russon P, Miles C, D'Alfonso S, Alvarez-Jimenez M (2019). Development of a moderated online intervention to treat social anxiety in first-episode psychosis. Front Psychiatry.

[12] Rice S, Robinson J, Bendall S, Hetrick S, Cox G, Bailey E, Gleeson J, Alvarez-Jimenez M (2016). Online and social media suicide prevention interventions for young people: a focus on implementation and moderation. J Can Acad Child Adolesc Psychiatry.

[13] Valentine L, McEnery C, D’Alfonso S, Phillips J, Bailey E, Alvarez-Jimenez M (2019). Harnessing the potential of social media to develop the next generation of digital health treatments in youth mental health. Curr Treat Options Psych.

[14] Nowland R, Necka EA, Cacioppo JT (2018). Loneliness and social internet use: pathways to reconnection in a digital world?. Perspect Psychol Sci.

[15] Maher CA, Lewis LK, Ferrar K, Marshall S, de Bourdeaudhuij I, Vandelanotte C (2014). Are health behavior change interventions that use online social networks effective? A systematic review. J Med Internet Res.

[16] Torous J, Nicholas J, Larsen ME, Firth J, Christensen H (2018). Clinical review of user engagement with mental health smartphone apps: evidence, theory and improvements. Evid Based Ment Health.

[17] D'Alfonso S, Phillips J, Valentine L, Gleeson J, Alvarez-Jimenez M (2019). Moderated online social therapy: viewpoint on the ethics and design principles of a web-based therapy system. JMIR Ment Health.

[18] Mohr DC, Cuijpers P, Lehman K (2011). Supportive accountability: a model for providing human support to enhance adherence to ehealth interventions. J Med Internet Res.

[19] Merriam SB (2015). Qualitative Research: A Guide to Design and Implementation.

[20] Saunders B, Sim J, Kingstone T, Baker S, Waterfield J, Bartlam B, Burroughs H, Jinks C (2018). Saturation in qualitative research: exploring its conceptualization and operationalization. Qual Quant.

[21] Smith JA, Osborne M, Breakwell G (2004). Interpretative phenomenological analysis. Doing Social Psychology Research.

[22] McGaha KK, D’Urso PA (2019). A non-traditional validation tool: using cultural domain analysis for interpretive phenomenology. Int J Soc Res Methodol.

[23] Morse JM (2015). Critical analysis of strategies for determining rigor in qualitative inquiry. Qual Health Res.

[24] Leggatt M, Woodhead G (2016). Family peer support work in an early intervention youth mental health service. Early Interv Psychiatry.

[25] Kilpatrick E, Keeney S, McCauley C (2017). Tokenistic or genuinely effective? Exploring the views of voluntary sector staff regarding the emerging peer support worker role in mental health. J Psychiatr Ment Health Nurs.

[26] Deci E, Ryan R, Wright JD (2015). Self-determination theory. International Encyclopedia of the Social & Behavioral Sciences.

[27] Smith W, Ploderer B, Wadley G, Webber S, Borland R (2017). Trajectories of Engagement and Disengagement with a Story-Based Smoking Cessation App. Proceedings of the 2017 CHI Conference on Human Factors in Computing Systems.

[28] Schueller SM, Stiles-Shields C, Yarosh L (2017). Online treatment and virtual therapists in child and adolescent psychiatry. Child Adolesc Psychiatr Clin N Am.

